# Identification of mitochondria-related genes associated with anesthetics in patients undergoing off-pump coronary artery bypass grafting surgery

**DOI:** 10.3389/fsurg.2025.1515732

**Published:** 2025-07-09

**Authors:** Yuhu Zhang, Xinjian Li, Tingru Sun

**Affiliations:** ^1^ Department of Anesthesiology Medicine, The First Hospital of Zibo, Zibo, China; ^2^ Department of Cardiovascular Medicine, The First Hospital of Zibo, Zibo, China

**Keywords:** heart surgery, anesthetics, propofol, sevoflurane, biomarker

## Abstract

**Background:**

Anesthetics have been reported to play a protective role in the heart during surgery. This study aimed to identify mitochondrial-related genes (Mito-RGs) involved in sevoflurane- and propofol-induced anesthesia in patients undergoing off-pump coronary artery bypass grafting (OPCABG) surgery.

**Methods:**

The GSE4386 dataset, which contains atrial samples obtained from patients receiving sevoflurane or propofol during OPCABG, was downloaded from GEO database for differential expression analysis and immune cell infiltration analysis between the pre-operative and post-surgery groups. Furthermore, to model the ischemia-reperfusion injury encountered during cardiac surgery, we established an *in vitro* hypoxia/reoxygenation (H/R) model and investigated the effects of sevoflurane and propofol on the expression of hub genes in cardiomyocytes subjected to H/R injury.

**Results:**

In this study, we identified a total of 11 common Mito-RGs that were influenced by sevoflurane and propofol during OPCABG. Furthermore, a PPI network of these genes was constructed using STRING, followed by the application of the MCODE and cytoHubba plug-ins to further identify hub genes within the network. Our analysis revealed that MCL1, RGS2, PPP1R15A, and MAFF may be the hub Mito-RGs associated with anesthetics. In the post-surgery group, the levels of these genes were negatively correlated with pro-inflammatory M1 macrophages. Notably, compared to pre-OPCABG levels, both sevoflurane and propofol significantly upregulated the expressions of these four hub genes in atrial samples following OPCABG. Furthermore, RT-qPCR and western blot analyses validated that both sevoflurane and propofol can upregulate the expression of Maff, Ppp1r15a, Rgs2, and Mcl1 in H9C2 cardiomyocytes following H/R injury.

**Conclusion:**

Collectively, these four genes may be linked to the potential cardioprotective effects of anesthetics during OPCABG, which could facilitate further research into the underlying mechanisms and contribute to the development of a more comprehensive and effective anesthesia protocol.

## 1 Introduction

Cardiovascular disease (CVD), including coronary artery disease (CAD) and stroke, is the leading cause of death in China (1, 2). Off-pump coronary artery bypass grafting (OPCABG) has emerged as a significant innovation in the surgical treatment of CAD (3–5). This technique allows surgeons to perform bypass grafting without stopping the heart, thus avoiding the need for extracorporeal circulation (6, 7). However, during the operative period, patients necessitate general anesthesia to ensure painlessness throughout the surgical procedure. Anesthesia not only maintains hemodynamic stability, but also protects myocardial function, prevents perioperative myocardial ischemia (8). Consequently, anesthetics are essential in cardiac surgery and significantly influence the patient's postoperative recovery and cardioprotection (9, 10). Sevoflurane, an inhalational anesthetic, and propofol, an intravenous anesthetic, are two commonly used agents that serve distinct roles in cardiac surgery (11). Both sevoflurane and propofol have been reported to exert cardioprotective effects (12–14). Compared to propofol, sevoflurane is believed to provide a more substantial cardioprotective effect during cardiac surgery (15, 16). Meanwhile, propofol may decrease the incidence of postoperative nausea and cognitive dysfunction compared to sevoflurane (15, 16). Generally, anesthesiologists select the appropriate anesthetic agent and dosage based on the specific requirements of the procedure and the individual patient's condition (17). Thus, understanding the protective molecular mechanisms of anesthetics during cardiac surgery is crucial for identifying molecular markers that could aid in mechanism research and the development of a more comprehensive and effective anesthesia protocol.

Mitochondria are particularly abundant in cardiac tissue, and the dysfunction of mitochondria is believed to significantly contribute to cardiac pathology (18). Enhancing mitochondrial biogenesis and maintaining mitochondrial homeostasis may help mitigate cardiomyocyte injury (19). Ollitrault et al. reported that mitochondrial dysfunction is associated with poor outcomes following CABG surgery (20). Identification of mitochondria-related genes (Mito-RGs) associated with the protective effects of anesthetic presents an opportunity to enhance anesthetic efficacy, leading to improved outcomes for patients undergoing CABG.

In this study, we analyzed the impact of the anesthetics on gene expression in patients undergoing OPCABG, utilizing the Gene Expression Omnibus (GEO) database (GSE4386 dataset), and with the aim of identifying potential biomarkers that may be related to the protective effects of anesthetics. Through comprehensive bioinformatics analysis, we identified four hub Mito-RGs-MCL1, RGS2, PPP1R15A, and MAFF-that were significantly associated with anesthetic exposure. Existing literature strongly supports the cardioprotective relevance of MCL1 and RGS2, as their downregulation has been linked to cardiac dysfunction (21, 22). While PPP1R15A and MAFF have been implicated in various pathological conditions (23, 24), their specific functions in cardiac physiology remain to be elucidated. Our results showed that these four genes may serve as therapeutic targets for improving cardiac outcomes in surgical patients. Meanwhile, our findings may provide novel insights into potential molecular mechanisms underlying anesthetic-mediated cardioprotection.

## 2 Materials and methods

### 2.1 Data collection

The data in the GSE4386 dataset was obtained from the Gene Expression Omnibus (GEO, https://www.ncbi.nlm.nih.gov/geo/) database. This dataset comprises 40 atrial samples collected from 10 patients who underwent off-pump CABG using sevoflurane gas anesthesia and 10 patients who received intravenous anesthesia with propofol. These samples were collected from the atrial tissues at both the beginning and end of surgery, and categorized into four groups: I: pre-OPCABG groups [a: pre-OPCABG_sevoflurane (Pre_sevoflurane) and b: pre-OPCABG_propofol (Pre_propofol)]; II: post-OPCABG groups [c: post-OPCABG_sevoflurane (Post_sevoflurane) and d: post-OPCABG_propofol (Post_propofol).

Mitochondrial-related genes (Mito-RGs) are defined as those genes that encode proteins localized within the mitochondria, encompassing all proteins found in the mitochondrial membrane, matrix, cristae, and associated with the membranes of mitochondria-related endoplasmic reticulum. A total of 1,735 Mito-RGs were collected from the MitoCarta 3.0 database and 23 cell component gene sets related to mitochondria in MSigDB (http://software.broadinstitute.org/gsea/msigdb) (25). These 1,735 unique genes were screened as Mito-RGs, and are listed in Supplementary Table S1.

The transcription factor (TF) gene sets were downloaded from the JASPAR database (https://jaspar.genereg.net/).

### 2.2 Differential expression analysis

Using the limma package (version 3.56.2) in R language, the expression data were presented as log transformed fold-change (log2FC) (26). Meanwhile, the false discovery rate (FDR, Benjamini-Hochberg method) method was employed to adjust *p*-values for multiple hypothesis testing. Differentially expressed genes (DEGs) between two groups were identified based on the criteria of |Log2FC| > 1 and FDR < 0.05.

### 2.3 Functional analysis

Utilizing the clusterProfiler package (version 4.8.3) in R language, Gene Ontology (GO) enrichment analysis, including Biological Processes (BP), Molecular Functions (MF), and Cellular Components (CC) terms, as well as Kyoto Encyclopedia of Genes and Genomes (KEGG) pathway enrichment analysis were conducted (27). Gene Set Enrichment Analysis (GSEA, http://www.broadinstitute.org/gsea/index.jsp) was performed based on KEGG pathway annotations. Pathways that met an adjusted *p*-value (Benjamini-Hochberg method) threshold of less than 0.05 were deemed significantly enriched.

### 2.4 Protein-protein interaction (PPI) network and hub genes identification

The interaction relationships between proteins and their corresponding interaction scores (combined_score) were predicted using the STRING online database (https://string-db.org/, version 12.0). The PPI network was constructed and visualized using the Cytoscape software (version 3.10.2), and key subnetworks were identified using the Molecular Complex Detection (MCODE). Subsequently, hub genes were determined via cytohubba plug-in using five algorithms, including Degree, Edge Percolated Component (EPC), Neighborhood Component Centrality (MNC), Maximum Neighborhood Component Centrality (DMNC), and Maximum Clique Centrality (MCC).

### 2.5 Analysis of immune cell infiltration

The relative proportions of 22 immune cell types within the sample were determined using the CIBERSORT software (28), a deconvolution algorithm that estimates relative proportions of 22 immune cell types from gene expression data (summing to 1 per sample). Additionally, the abundance of the 22 specific immune cell types was calculated using the ssGSEA algorithm.

### 2.6 Construction of miRNA-mRNA network

The relationship between miRNA and mRNA was obtained from the ENCORI database (https://rnasysu.com/encori/index.php). The miRNA-mRNA network was visualized using the Cytoscape software (version 3.10.2).

### 2.7 Prediction of transcription factor (TF) binding sites of hub genes

The 2,000 base pair (bp) upstream region and the 200 bp downstream region of the gene start sites were downloaded from the UCSC Genome Browser (http://genome.ucsc.edu/). Next, the motif file corresponding to the transcription factor was acquired from the JASPAR database (https://jaspar.genereg.net/). The online tool FIMO (https://meme-suite.org/meme/tools/fimo) was utilized to predict the presence of the TF binding motif within the gene promoter region and to obtain the binding region along with the binding score.

### 2.8 Cell culture and treatment

The H9C2 rat embryo cardiomyocyte cell line was maintained in DMEM medium supplemented with 10% fetal bovine serum and 1% penicillin/streptomycin and culture with 5% CO_2_ at 37°C. A hypoxia/reoxygenation (H/R) model was created by exposing H9C2 cells to a environment without oxygen (95% N_2_ and 5% CO_2_) for 6 h. Following this, the cells were returned to standard conditions (95% air and 5% CO_2_) for an additional 6 h. For cell treatment, cells were treated with 2% sevoflurane (Solarbio) or 25 µmol/L propofol (Solarbio) and cultured in standard conditions for 1 h prior to H/R.

### 2.9 Enzyme-linked immunosorbent assay (ELISA)

The Rat Lactate Dehydrogenase (LDH) ELISA Kit (JL13677, JONLNBIO) and malondialdehyde (MDA) ELISA Kit (JL53632, JONLNBIO) were utilized for assessing LDH and MDA concentrations in H9C2 cells, respectively.

### 2.10 Cell counting kit-8 (CCK-8) assay

H9C2 cells were plated in a 96-well and allowed to culture overnight. Following the treatments, 10 μl of CCK-8 reagent was added to each well for a duration of 2 h. Then, the absorbance for each well was quantified using a microplate reader at a wavelength of 450 nm.

### 2.11 Real time-quantitative polymerase chain reaction (Rt-qPCR)

Total RNA extraction from cells was performed using the TRNzol Universal total RNA extraction reagent (DP424, TIANGEN), followed by reverse transcription into cDNA with the Evo M-MLV Reverse Transcription Premix Kit (AG11728, Accurate Biology). Next, the mRNA levels of Mcl1, Rgs2, Ppp1r15a, and Maff were assessed via RT-qPCR utilizing the SuperStar Universal SYBR Master Mix (CWBIO), with Gapdh serving as the endogenous control. Meanwhile, gene mRNA levels were quantified using the 2^−*ΔΔ*Ct^ method. The primers used as follows: Mcl1, forward: 5’-AAAGGCGGCTGCATAAGTC-3’ and reverse: 5’-TGGCGGTATAGGTCGTCCTC-3’; Rgs2, forward: 5’-GAGAAAATGAAGCGGACACTCT-3’ and reverse: 5’-GCAGCCAGCCCATATTTACTG-3’; Ppp1r15a, forward: 5’-GAGGGACGCCCACAACTTC-3’ and reverse: 5’-GAGGGAGGAGGTTACCAGAGA-3’; Maff, forward: 5’-CAGCCCTACAGCAACAGCA-3’ and reverse: 5’-GCTGGGTCAGAGGCATAGG-3’; Gapdh, forward: 5’-AGGTCGGTGTGAACGGATTTG-3’ and reverse: 5’-GGGGTCGTTGATGGCAACA-3’.

### 2.12 Western blot

The concentration of protein was assessed utilizing the BCA protein quantification kit (CWBIO). Following this, the proteins were separated using 10% SDS-PAGE and subsequently transferred onto PVDF membranes. The membranes underwent an overnight incubation at 4°C with primary antibodies such as anti-Mcl1 (16225-1-AP, Proteintech), anti-Rgs2 (ab36561, Abcam), anti-Ppp1r15a (10449-1-AP, Proteintech), anti-Maff (12771-1-AP, Proteintech), and anti-Gapdh (60004-1-Ig, Proteintech), followed by incubation with corresponding secondary antibody. The detection of the blots was performed with the BeyoECL Moon kit.

### 2.13 Statistical analysis

The Wilcoxon rank sum test was employed to compare differences across various groups. Pearson correlation analysis was conducted using the R language. *P*-value less than 0.05 was considered statistically significant. All statistical analyses were performed using R software (version 4.3.3).

For cell-based experiments, one-way analysis of variance was employed for multiple comparisons. The data are shown as mean ± standard deviation. Each experiment was independently repeated at least three times. A *P*-value of less than 0.05 indicates statistical significance.

## 3 Results

### 3.1 Identification of mitochondrial-related DEGs (DE-Mito-RGs) affected by anesthetics during OPCABG

**Figure 1:**
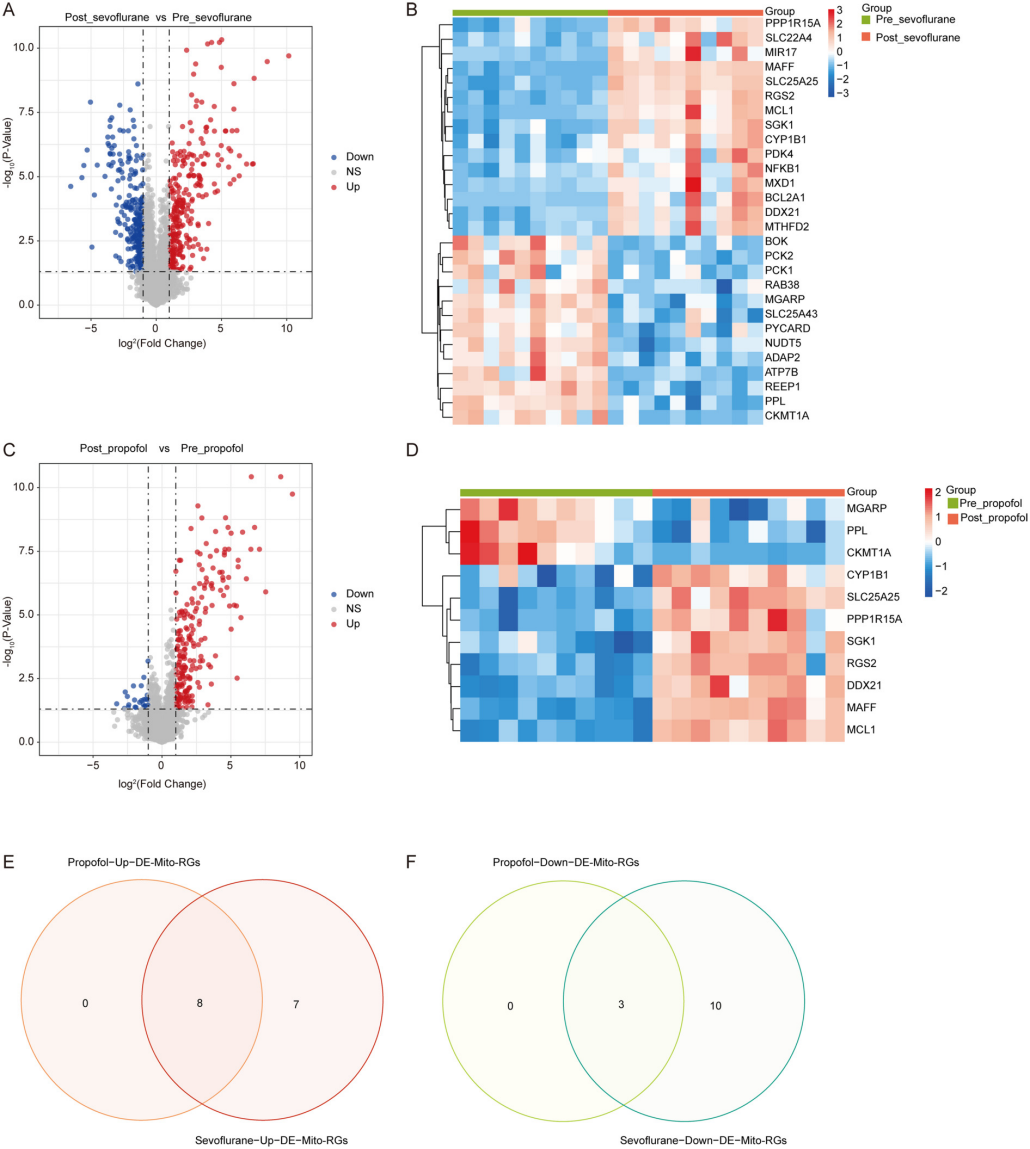
Identification of mitochondrial-related DEGs (DE-mito-RGs) affected by anesthetics during OPCABG. **(A)** Volcano plot of DEGs between Post_sevoflurane and Pre_sevoflurane groups in the GSE4386 dataset. The *x*-axis represents log2(Fold Change), with dashed reference lines at −1 and 1. The *y*-axis represents -log10(*P*-Value), with a dashed reference line at -log10(0.05). Red dots indicate upregulated genes, while blue dots represent downregulated genes. **(B)** Heatmap of DE-Mito-RGs between Post_sevoflurane (red color) and Pre_sevoflurane (green color) groups in the GSE4386 dataset. **(C)** Volcano plot of DEGs between Post_propofol and Pre_propofol groups in the GSE4386 dataset. The *x*-axis represents log2(Fold Change), with dashed reference lines at −1 and 1. The *y*-axis represents -log10(*P*-Value), with a dashed reference line at -log10(0.05). Red dots indicate upregulated genes, while blue dots represent downregulated genes. **(D)** Heatmap of DE-Mito-RGs between Post_propofol (red color) and Pre_propofol (green color) groups in the GSE4386 dataset. **(E,F)** Venn map of eight common **(E)** upregulated and **(F)** three downregulated DE-Mito-RGs between two comparison groups: Post_sevoflurane vs. Pre_sevoflurane group and Post_propofol vs. Pre_propofol group.

The R language “limma” package was employed to screen DEGs between Pre_sevoflurane and Post_sevoflurane groups, as well as between Pre_propofol and Post_propofol groups. Notably, compared to the Pre_sevoflurane group, the levels of 299 genes were upregulated and the levels of 278 genes were downregulated in the Post_sevoflurane group (Figure 1A; Supplementary Table S2). Among the 577 DEGs, there were 15 upregulated Mito-RGs and 13 downregulated Mito-RGs in the Post_sevoflurane group compared to the Pre_sevoflurane group (Figure 1B).

Significantly, compared to the Pre_propofol group, the levels of 212 genes were upregulated and the levels of 23 genes were downregulated in the Post_propofol group (Figure 1C; Supplementary Table S2). Among the 235 DEGs, there were 8 upregulated Mito-RGs and 3 downregulated Mito-RGs in the Post_propofol group compared to the Pre_propofol group (Figure 1D).

Next, to screen DE-Mito-RGs affected by anesthetics during OPCABG, a Venn diagram was performed to identify common genes between the two comparison groups: Post_sevoflurane vs. Pre_sevoflurane and Post_propofol vs. Pre_propofol. The analysis revealed 8 common upregulated and 3 common downregulated Mito-RGs in the post-sevoflurane/propofol group compared to pre-sevoflurane/propofol group (Figures 1E,F; Supplementary Table S2). These 11 genes were considered as candidate DE-Mito-RGs affected by anesthetics during OPCABG.

### 3.2 Functional analysis

**Figure 2:**
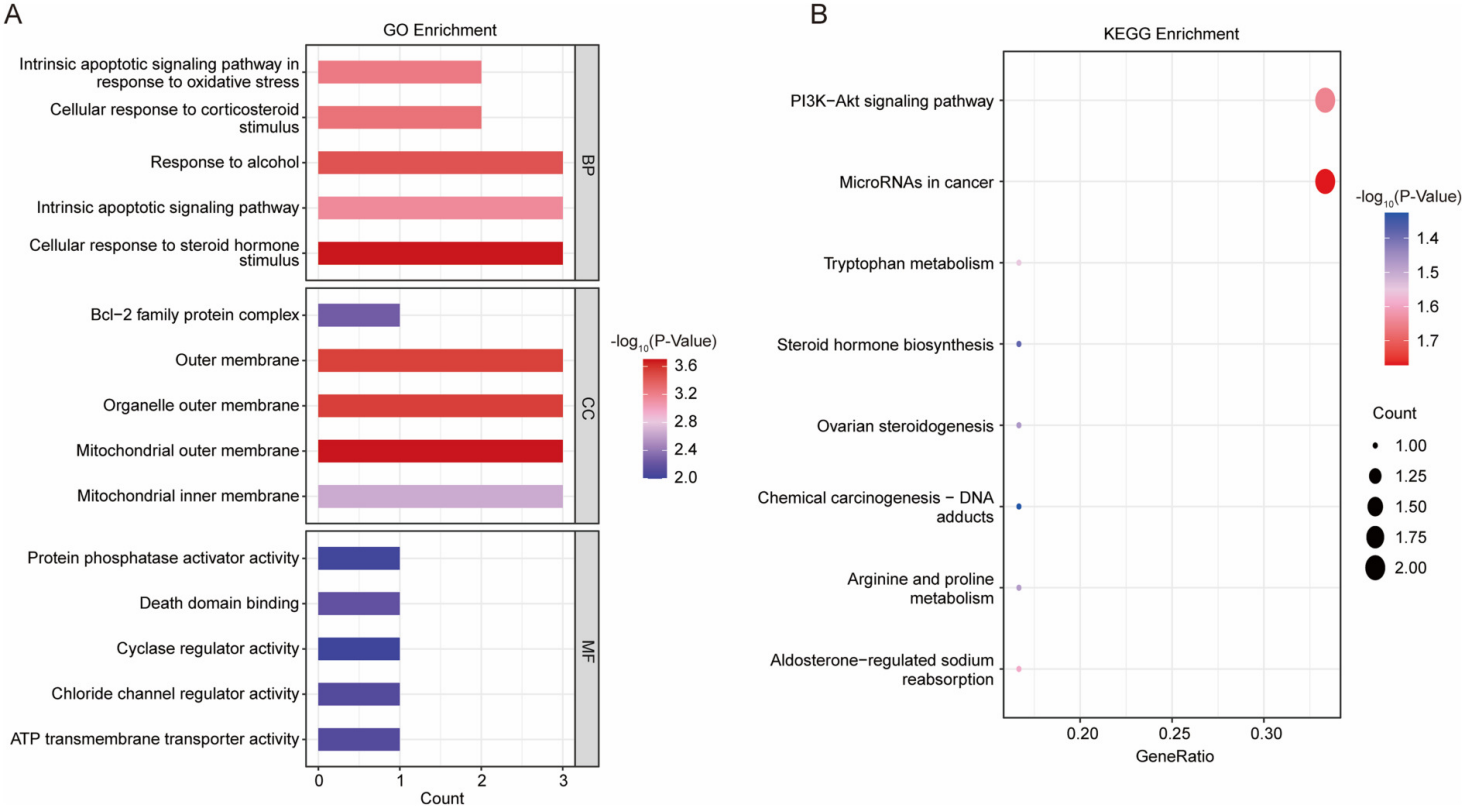
Functional analysis on 11 DE-mito-RGs. **(A)** The top 5 GO terms in BP, CC, and MF of GSE4386 dataset. **(B)** Eight enriched KEGG pathways of GSE4386 dataset.

To gain a comprehensive understanding of the 11 DE-Mito-RGs, we conducted GO and KEGG functional enrichment analyses. These DE-Mito-RGs were mainly related to 294 GO terms, including 244 BP (e.g., “Cellular response to steroid hormone stimulus”), 13 CC (e.g., “Mitochondrial outer membrane”) and 37 MF terms (e.g., “ATP transmembrane transporter activity”) (Supplementary Table S3). The top 5 GO terms in BP, CC, and MF were illustrated in Figure 2A. Meanwhile, these genes were associated with 8 KEGG pathways, including “PI3K-Akt signaling pathway”, “Aldosterone-regulated sodium reabsorption”, “Tryptophan metabolism”, “Arginine and proline metabolism” and “Steroid hormone biosynthesis” (Figure 2B; Supplementary Table S3).

### 3.3 Identification of hub DE-Mito-RGs

**Figure 3:**
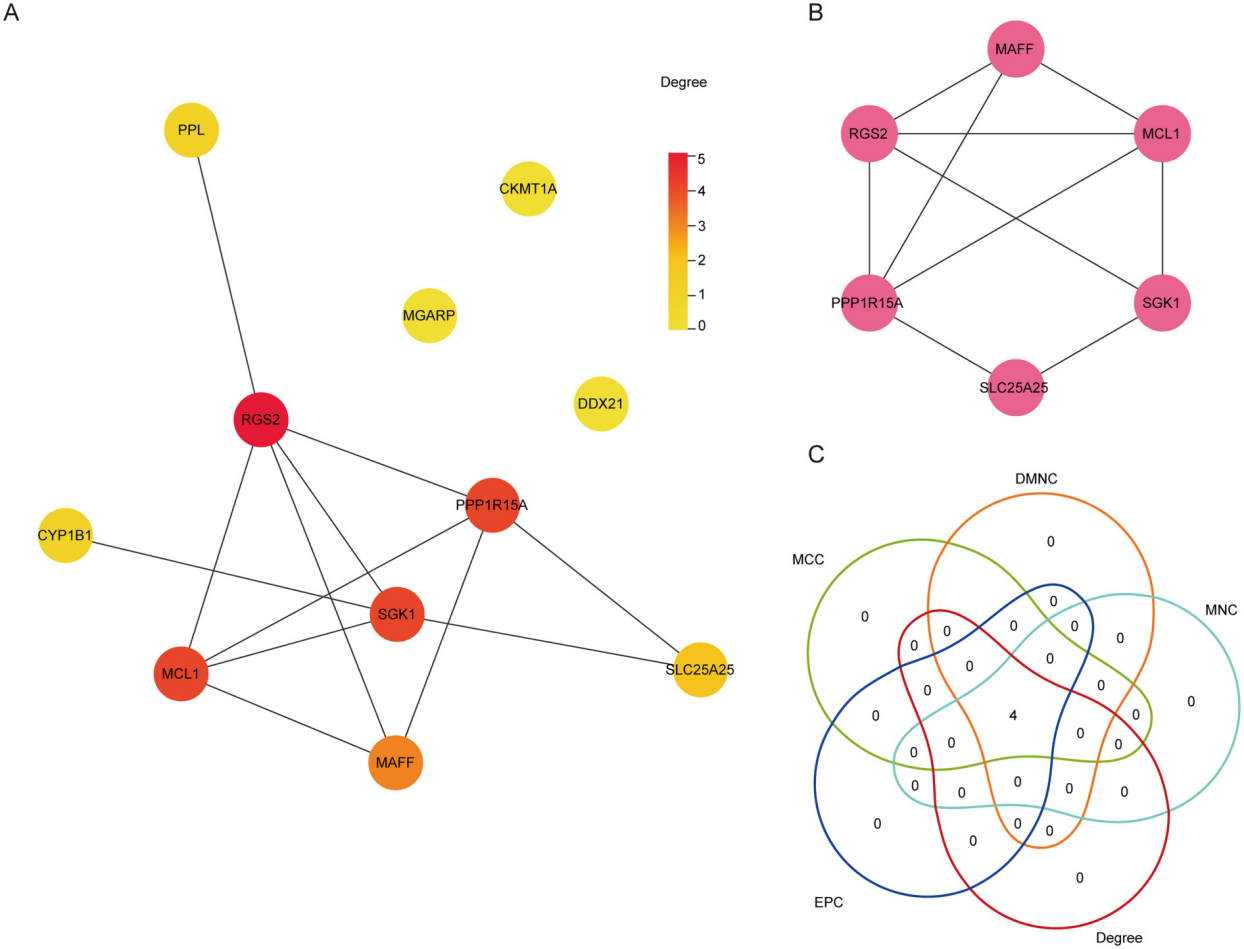
Identification of hub DE-mito-RGs. **(A)** The PPI network diagram showing the interaction among the 11 DE-Mito-RGs. The color of each node represents the degree of freedom of gene in the network. The closer the distance between two proteins, the easier the binding between proteins. **(B)** The key subnetworks identified by Cluster, including six genes. **(C)** Venn diagram showing the four hub genes obtained by five algorithms used in Cytohubba.

The interactions between DE-Mito-RGs at the protein levels were analyzed using the String online database. Then, a PPI network was constructed, comprising eleven nodes (eleven genes) and twelve edges with an average number of neighbors of three (Figure 3A). Next, the key sub-network exhibiting high clustering was generated using the MCODE plugin of Cytoscape software, which included six nodes (six genes) and attained a cluster score of four (Figure 3B). This suggests that these genes may exhibit closely interrelated functional associations. Thereafter, the six genes in the sub-network were further extracted using the cytoHubba plug-in, revealing that MCL1, RGS2, PPP1R15A, and MAFF consistently ranked in the top four across all five evaluated algorithms (Figure 3C). Thus, these four genes (MCL1, RGS2, PPP1R15A, and MAFF) may be considered as significant hub Mito-RGs.

**Figure 4:**
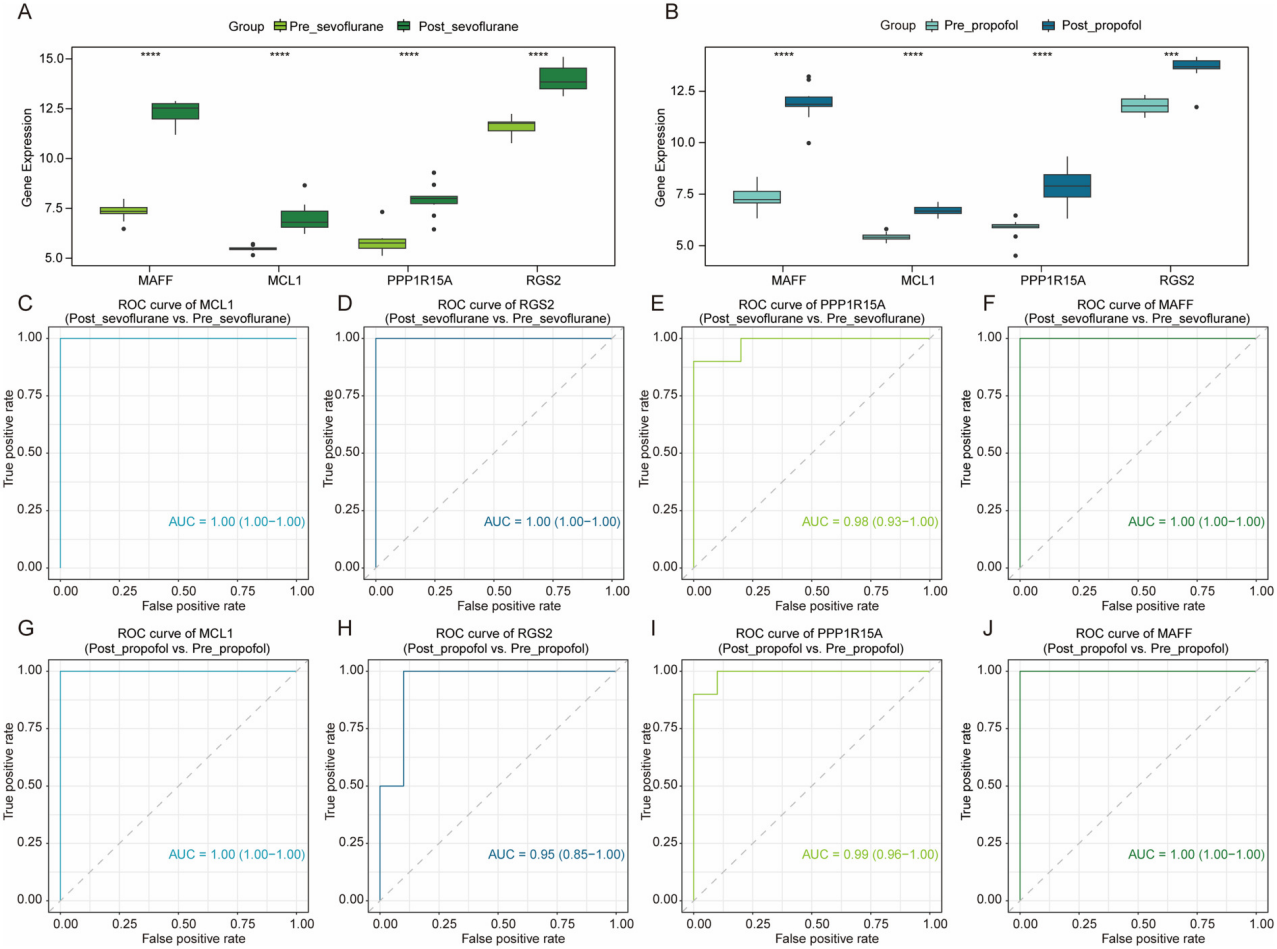
The potential value of hub DE-mito-RGs for anesthetics-induced protection during OPCABG. **(A)** Box plots showing the expression levels of MCL1, RGS2, PPP1R15A, and MAFF between Post_sevoflurane (dark green) and Pre_sevoflurane (light green) groups. **(B)** Box plots showing the expression levels of MCL1, RGS2, PPP1R15A, and MAFF between Post_propofol (dark blue) and Pre_propofol (light blue) groups. **(C–F)** ROC curve of MCL1 (AUC = 1), RGS2 (AUC = 1), PPP1R15A (AUC = 0.98), and MAFF (AUC = 1) in Post_sevoflurane vs. Pre_sevoflurane comparison group. **(G–J)** ROC curve of MCL1 (AUC = 1), RGS2 (AUC = 0.95), PPP1R15A (AUC = 0.99), and MAFF (AUC = 1) in Post_propofol vs. Pre_propofol comparison group.

As shown in Figures 4A,B, compared to pre-OPCABG, both sevoflurane and propofol significantly upregulated the mRNA levels of MCL1, RGS2, PPP1R15A, and MAFF in atrial samples following OPCABG in the GSE4386 dataset. Next, to assess the potential of these four genes as cardioprotective biomarkers, we constructed the ROC curve of logistic regression. As shown in Figures 4C–J, the AUC values for all four genes were all greater than 0.9 in both comparison groups: Post_sevoflurane vs. Pre_sevoflurane group and Post_propofol vs. Pre_propofol group, indicating a very high level of accuracy. Thus, these four genes may serve as novel anesthetic-mediated protective biomarkers during cardiac surgery.

### 3.4 Immune cell infiltration analysis

**Figure 5:**
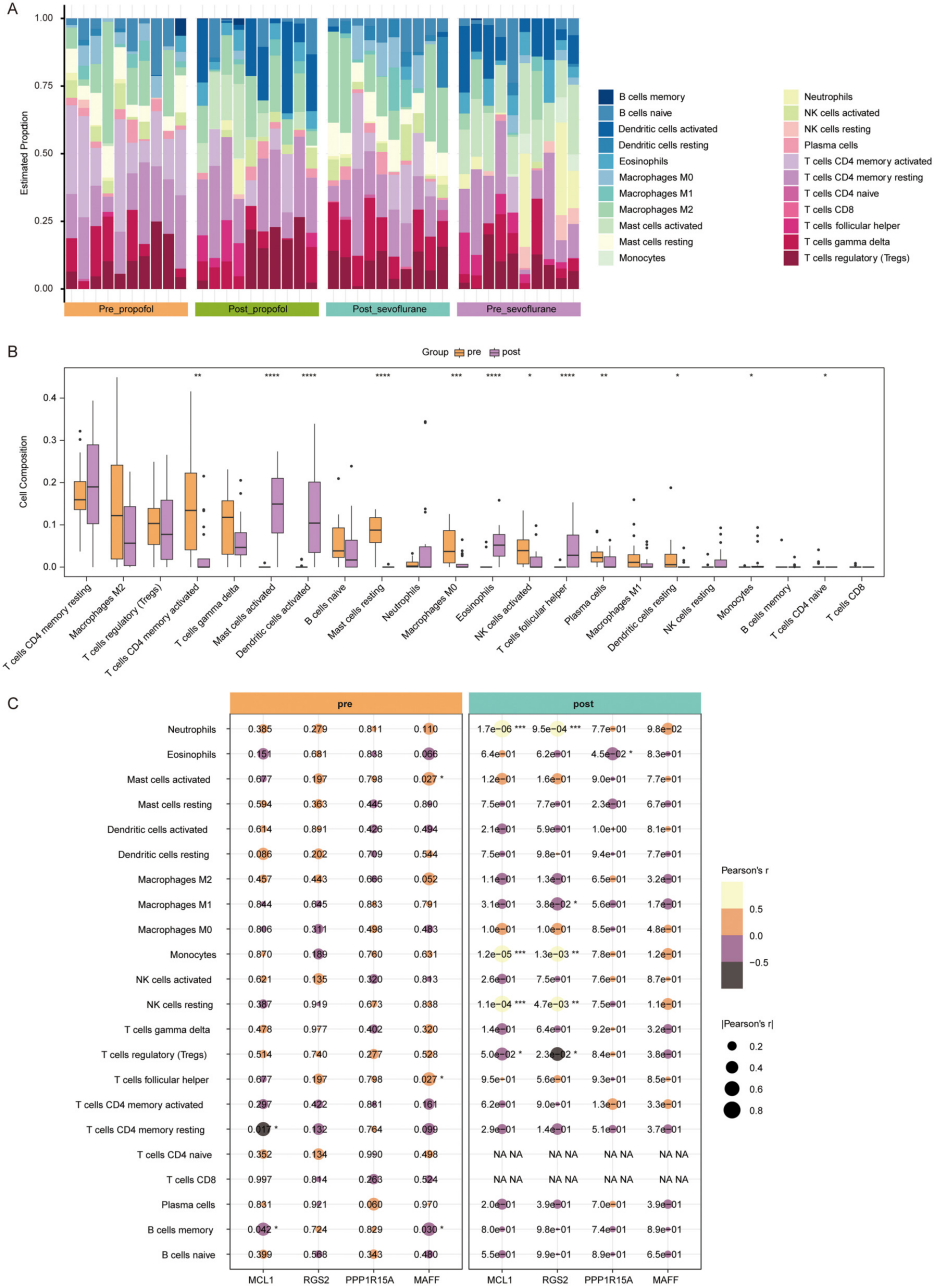
Immune cell infiltration analysis. **(A)** The infiltration proportions of 22 types of immune cells in all samples of Post_propofol, Pre_propofol, Post_propofol and Pre_propofol groups. The *x*-axis represents samples, with bar labels indicating groups. The *y*-axis shows the estimated infiltration proportion, while bar colors correspond to immune cell types. **(B)** Box plots showing levels of 22 types of immune cells between pre-OPCABG (merged Pre_propofol and Pre_propofol) and post-OPCABG groups (merged Post_propofol and Post_propofol). **(C)** Correlation heat map showing the correlation between MCL1, RGS2, PPP1R15A, or MAFF and 22 types of immune cells in pre-OPCABG (merged Pre_propofol and Pre_propofol) and post-OPCABG (merged Post_propofol and Post_propofol) groups.

It has been demonstrated that immune cells play a crucial role in maintaining atrial stability (29). Thus, we proceeded to analyze the immune cell infiltration levels in all samples using CIBERSORT (Figure 5A). In patients anesthetized with sevoflurane, the levels of M2 macrophages, M1 macrophages, activated CD4 memory T cells, resting mast cells, resting dendritic cells, activated NK cells and plasma cells were markedly reduced, and the levels of activated mast cells, activated dendritic cells, eosinophils, follicular helper T cells, and resting NK cells were notably increased in atrial samples after surgery (Supplementary Figure S1A). In patients anesthetized with sevoflurane, the levels of activated CD4 memory T cells, resting mast cells, M0 macrophages were greatly decreased, and activated dendritic cells, activated mast cells, eosinophils, follicular helper T cells were significant increased in atrial samples after surgery (Supplementary Figure S1B).

Next, samples from two anesthesia types were combined to analyze anesthetics-induced changes in immune cell infiltration. Compared to the pre-OPCABG group, the levels of activated CD4 memory *T* cells, naive CD4 *T* cells, M0 macrophages, resting mast cells, activated NK cells, plasma cells and resting dendritic cells were decreased, and the levels of follicular helper T cells, activated mast cells, activated dendritic cells, eosinophils and monocytes were elevated in the post-OPCABG group (Figure 5B).

Furthermore, we analyzed the correlation between hub DE-Mito-RGs and 22 types of immune cells (Figure 5C). In post-OPCABG group, MCL1, RGS2, PPP1R15A, and MAFF levels were found to be negatively correlated with M1 macrophages (Figure 5C).

### 3.5 Construction of hub DE-Mito-RGs-miRNAs regulatory network

**Figure 6:**
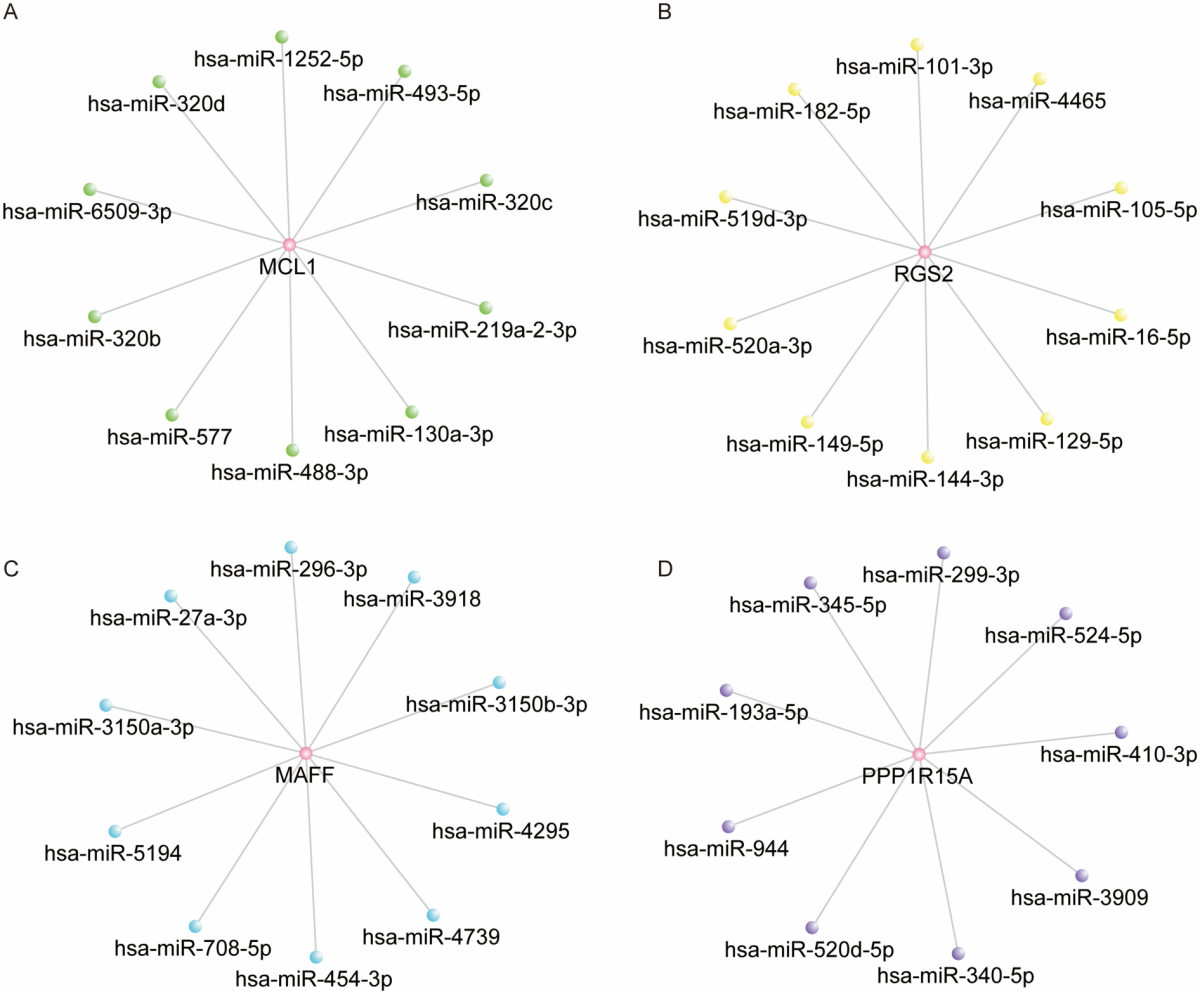
Construction of hub DE-mito-RGs-miRNAs regulatory network. **(A)** The network of MCL1 and top 10 predicted miRNAs (ranked by the TDMD score). **(B)** The network of RGS2 and top 10 predicted miRNAs (ranked by the TDMD score). **(C)** The network of MAFF and top 10 predicted miRNAs (ranked by the TDMD score). **(D)** The network of PPP1R15A and 9 predicted miRNAs.

ENCORI database was conducted to predict the upstream miRNAs associated with four hub genes. As shown in Supplementary Table S4, the data showed the potential relationship between MCL1 and 247 miRNAs, RGS2 and 59 miRNAs, PPP1R15A and 9 miRNAs, MAFF and 67 miRNAs. The network for MCL1, RGS2, MAFF and their top 10 predicted miRNAs were shown in Figures 6A–C. The network for PPP1R15A and its 9 miRNAs were shown in Figure 6D.

### 3.6 Construction of hub DE-Mito-RGs-TFs regulatory network

**Figure 7:**
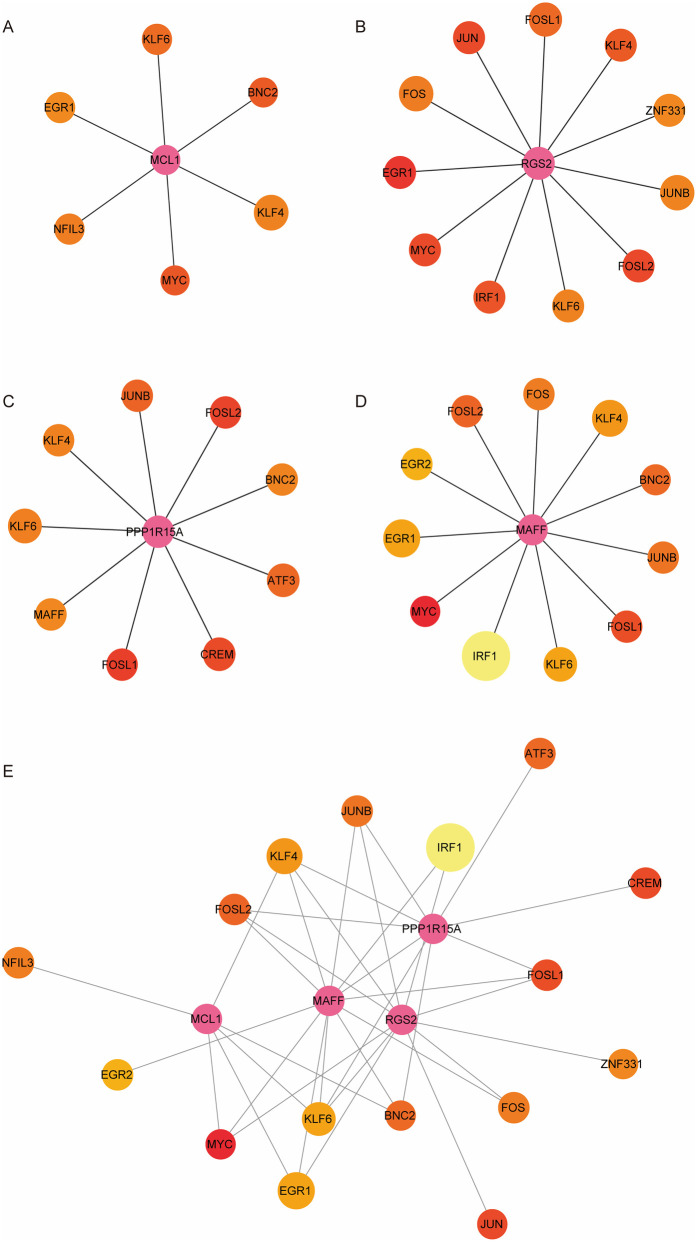
Construction of hub DE-mito-RGs-TFs regulatory network. **(A)** The network of MCL1 and six TFs. **(B)** The network of RGS2 and eleven TFs. **(C)** The network of PPP1R15A and nine TFs. **(D)** The network of MAFF and eleven TFs. **(E)** The network of hub genes (MCL1, RGS2, PPP1R15A, and MAFF) and TFs. The size of each node represents the number of binding regions between TFs and the hub gene promoter. The pink node represents the hub gene. The colors of the other nodes represent the average scores of the binding regions where TFs binds to hub gene promoter.

To further explore the regulatory network of four hub genes, we identified a total of 25 TFs that were differentially expressed in both two comparison groups: Post_sevoflurane vs. Pre_sevoflurane group and Post_propofol vs. Pre_propofol group (Supplementary Table S5). Next, the binding ability of hub genes and 25 TFs was evaluated by the FIMO software, establishing a threshold of *p*-value < 0.0001 and q-value <0.05 to screen for potential binding regions. The results showed that six TFs may bind to the promoter region of MCL1; eleven TFs may bind to the promoter region of RGS2; nine TFs may bind to the promoter region of PPP1R15A; and eleven TFs may bind to the promoter region of MAFF (Figures 7A–D). Next, the TFs-hub gene regulatory network was constructed using Cytoscape. Additionally, as shown in Figure 7E; Supplementary Table S6, both KLF4 and KLF6 have the potential to bind to the promoter regions of MCL1, RGS2, PPP1R15A, or MAFF.

### 3.7 Sevoflurane and propofol upregulated maff, Ppp1r15a, Rgs2 and Mcl1 levels in cardiomyocytes exposed to H/R

**Figure 8:**
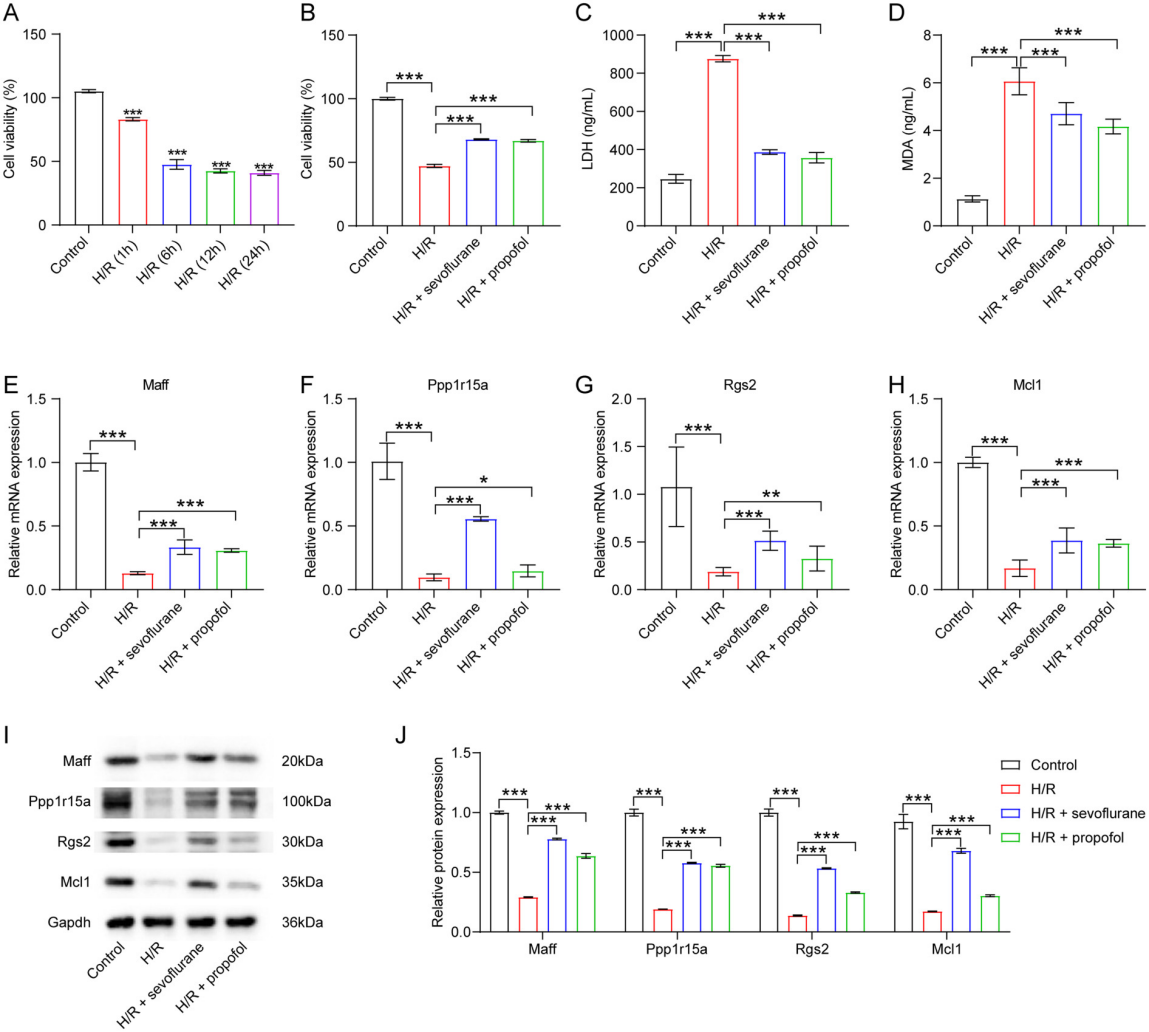
Sevoflurane and propofol upregulated maff, Ppp1r15a, Rgs2 and Mcl1 levels in cardiomyocytes exposed to H/R. **(A)** H9C2 cells were subjected to 6 h of hypoxia followed by 1, 6, 12 or 24 h of reoxygenation. Cell viability was evaluated using CCK-8 assay. **(B)** H9C2 cells were pre-treated with sevoflurane or propofol, and then exposed to 6 h of hypoxia/6 h of reoxygenation. Cell viability was evaluated using CCK-8 assay. **(C,D)** LDH and MDA levels in H9C2 cells were assessed by ELISA. **(E–H)** RT-qPCR and **(I–J)** western blot assays were employed to determine Maff, Ppp1r15a, Rgs2 and Mcl1 levels in H9C2 cells.

Research has demonstrated that OPCABG surgery can induce localized myocardial ischemia (30). To simulate and investigate the resulting cardiac injury *in vitro*, we established a H/R model using H9C2 cardiomyocytes (31). Compared to the control group, H9C2 cells subjected to 6 h of hypoxia followed by 6 h of reoxygenation exhibited an approximately 50% reduction in cell viability (Figure 8A). Thus, subsequent experiments employed this 6-hour hypoxia/6-hour reoxygenation protocol. Notably, H/R significantly decreased cell viability and increased LDH and MDA levels in H9C2 cells (Figures 8B–D). However, both sevoflurane and propofol treatment markedly improved cell viability as well as reduced MDA and LDH levels in H/R-stimulated H9C2 cells compared to the H/R group (Figures 8B–D), indicating that these anesthetics can mitigate H/R-induced cellular damage.

Further analysis revealed that H/R significantly downregulated the expression of Maff, Ppp1r15a, Rgs2, and Mcl1 in H9C2 cells (Figures 8E–J). Intriguingly, these effects were reversed by sevoflurane and propofol administration (Figures 8E–J). In summary, our findings demonstrate that both sevoflurane and propofol can upregulate Maff, Ppp1r15a, Rgs2, and Mcl1 expression in cardiomyocytes following H/R injury.

## 4 Discussion

Increasing evidence has shown that both inhalational and intravenous anesthetics have been demonstrated to have myocardial protective properties (32). Several studies indicate that the myocardial protective effects of sevoflurane or propofol are associated with their influence on mitochondria (9, 33). Zhang et al. demonstrated that propofol can inhibit hypoxia-induced cardiomyocyte apoptosis through upregulation of LRPPRC, a mitochondrial-associated protein (33). Meanwhile, Xie et al. reported that sevoflurane mitigates mitochondria damage in rat hearts subjected to myocardial ischemia/reperfusion injury (34). These findings suggest that mitochondria play a crucial role in myocardial injury. Thus, in this study, we mainly focused on the identification of Mito-RGs associated with anesthetics. Utilizing the GSE4386 dataset, we identified a total of 11 common Mito-RGs that were dysregulated between the pre- and post-surgery groups. Subsequently, a PPI network of these 11 genes was constructed using the STRING database and visualized by Cytoscape. Meanwhile, the MCODE and cytoHubba plug-ins of Cytoscape was employed to further screen hub genes within the network. The results showed that MCL1, RGS2, PPP1R15A, and MAFF may be the hub Mito-RGs related to anesthetics.

In this study, we observed that, compared to pre-OPCABG surgery, both sevoflurane and propofol significantly upregulated MCL1 and RGS2 mRNA levels in atrial samples following OPCABG. Several studies have demonstrated that MCL1 and RGS2 play significant roles in cardiac homeostasis and disease (21, 22). MCL1, an anti-apoptotic protein (35), is essential for cell survival and mitochondrial morphology (36). It has been shown that MCL1 is overexpressed in normal myocardium, and MCL1 deficiency in myocytes can lead to mitochondrial dysfunction (37). Furthermore, the downregulation of MCL1 is associated with cardiomyocyte death and cardiac dysfunction (21). Conversely, the activation of the PI3 K/Akt/GSK-3β/MCL1 pathway has been found to enhance cardioprotective effects in doxorubicin-stimulated cardiomyocytes (38). These findings suggest that elevated levels of MCL1 may confer cardioprotective benefits. Furthermore, growing evidence highlights mitochondrial dysfunction as an important pathological mechanism in various common disorders including cardiovascular diseases (39). MCL-1 has recently been shown to regulate mitochondrial energy metabolism by enhancing fatty acid β-oxidation (40). Importantly, upregulating fatty acid β-oxidation and improving mitochondrial function have demonstrated significant cardioprotective effects (41). Thus, our findings of anesthetic-induced MCL-1 upregulation in OPCABG patients may suggest a potential novel mechanism whereby these agents may confer myocardial protection through MCL-1-mediated metabolic modulation; however, this proposed mechanism requires further validation.

RGS2 is a GTPase activating protein (42), and its dysregulation has been linked to various human diseases (43). For instance, reduced expression of RGS2 may be related to the occurrence of mild cognitive impairment (43, 44). Meanwhile, mice deficient in RGS2 exhibit increased susceptibility to heart failure (22). Furthermore, RGS2 overexpression has been found to attenuate beta-adrenergic receptor-triggered cardiomyocyte hypertrophy by suppressing the activation of two signaling pathways, including ERK1/2 and Akt signalings (45), suggesting that RGS2 may have protective effects in the injured heart. Based on the established evidence, we hypothesize that RGS2-dependent regulation of these signaling pathways may contribute to anesthetic-mediated cardioprotection during OPCABG; however, this hypothesis requires further investigation.

Currently, there is no literature data demonstrating the roles of PPP1R15A and MAFF in heart function or structure. Aberrantly expressed PPP1R15A has been implicated in various diseases, including sepsis and pulmonary fibrosis. Jiang et al. indicated that PPP1R15A levels were reduced in a rat model of sepsis-associated acute lung injury (46). Ito et al. demonstrated that PPP1R15A, also known as GADD34, could inhibit inflammatory response and mitigate liver injury in a lipopolysaccharide-induced murine sepsis model through inhibiting macrophage activation (47). Additionally, a study by Monkley et al. reported that PPP1R15A was significantly decreased in the lung tissues of idiopathic pulmonary fibrosis patients, and PPP1R15A downregulation was capable of promoting lung fibroblast senescence (24). Meanwhile, PPP1R15A has also been shown to enhance cognitive function in Alzheimer's disease model mice (48). These findings suggest that elevated levels of PPP1R15A may confer a protective role across multiple diseases. Emerging evidence highlights the pivotal role of endoplasmic reticulum stress (ERS) in cardiac pathophysiology (49, 50). ERS serves as a critical mediator of myocardial injury, with pharmacological inhibition of ERS showing significant cardioprotective potential (49, 50). Jiang et al. demonstrated that PPP1R15A confers protection against sepsis-related lung injury via suppressing ERS (46). In this study, our results revealed that compared to pre-OPCABG surgery, both sevoflurane and propofol notably upregulated PPP1R15A mRNA levels in atrial samples following OPCABG. Thus, we propose a mechanistic hypothesis whereby these anesthetic agents may exert cardioprotection during OPCABG through a PPP1R15A-mediated attenuation of ERS; however, this hypothesis warrants further investigation.

MAFF has been implicated in a variety of cell biology processes, including apoptosis and inflammation (51, 52). For instance, Wang et al. found that overexpression of MAFF could suppress palmitic acid-induced early apoptosis in HUVECs (52). Additionally, Ibrahim et al. reported that MAFF could suppress the life cycle of the hepatitis B virus, thereby exerting an antiviral effect (53). Meanwhile, Lei et al. reported that MAFF was able to mitigate hepatic ischemia-reperfusion injury through activating CLCF1/STAT3 signaling pathway (54). STAT3 pathway has been found to play a role in protecting cardiomyocytes against hypoxia injury (55, 56). Furthermore, Cen et al. found that Nrf2-MAFF/ARE signaling play a role in maintaining mitochondrial hemostasis and improving endothelial injury (57). In this study, we found that compared to pre-OPCABG surgery, both sevoflurane and propofol notably upregulated MAFF mRNA levels in atrial samples following OPCABG. Thus, we speculated that MAFF may contribute to anesthetic-mediated cardioprotection through multiple mechanisms, such as preserving mitochondrial function and activating STAT3-dependent signaling; however, this hypothesis warrants further investigation.

To further explore the upregulation mechanisms of these four hub genes under general isoflurane/propofol anesthesia during OPCABG, we examined the upstream TFs associated with these four hub genes. Our analysis indicated that KLF4 may be linked to all four hub genes, and may potentially bind to their promoter regions. Previous studies have demonstrated that KLF4 plays a crucial role in enhancing cardiac function by maintaining mitochondrial homeostasis (58). Additionally, Li et al. reported that the downregulation of KLF4 could aggravate myocardial ischemia/reperfusion injury in mice through promoting mitochondrial fission (59). Furthermore, KLF4 has been identified as a transcriptional activator (60). Therefore, the upregulation of MCL1, RGS2, PPP1R15A, or MAFF may be associated with the upregulation of KLF4 under general isoflurane/propofol anesthesia during OPCABG. However, this hypothesis remains highly speculative and requires validation in future studies.

Topal et al. found that patients who developed atrial fibrillation following OPCABG exhibited a higher percentage of immune cells, including leukocytes and neutrophils, in their blood, as well as increased systemic immune inflammation, compared to those who did not develop atrial fibrillation (61). Additionally, perioperative inflammatory activation may be associated with the mortality of patients after OPCABG (62). Thus, immune cell infiltration may be related to the prognosis of patients undergoning OPCABG. Evidence indicates that sevoflurane contributes to macrophage M2 polarization, potentially promoting cancer progression (63, 64). In parallel, propofol has been demonstrated to enhance macrophage M2 polarization through the modulation of PPARγ/STAT3 signaling, thereby alleviating renal ischemia/reperfusion injury (65). Collectively, these findings suggest that both anesthetics have the capacity to influence macrophage polarization, specifically by promoting M2 macrophage polarization. Furthermore, elevating M2 macrophage polarization has been shown to improve cardiac function in cases of viral myocarditis (66). Conversely, reduced M1 macrophage polarization and diminished infiltration of Ly-6C + monocytes and neutrophils are associated with improved cardiac function (67). Meanwhile, the promotion of M1 macrophage polarization could lead to excessive inflammation and cardiac injury (68). Overall, these results imply that anesthetics may mitigate cardiac injury by modulating macrophage polarization. In this study, we found that in patients anesthetized with sevoflurane, the levels of M1 macrophages were markedly reduced in atrial samples after OPCABG. Furthermore, in the post-off-pump CABG group, MCL1, RGS2, PPP1R15A, and MAFF levels were negatively correlated with M1 macrophages. This indicates that low infiltration of M1 macrophages in atrial samples after OPCABG may be associated with the elevated levels of MCL1, RGS2, PPP1R15A, and MAFF induced by anesthetics. Significantly, Tan et al. demonstrated that MCL1 downregulation contributes to macrophages M1 polarization through upregulating the ASK1/MKK7/JNK signaling (69), suggesting a relationship between MCL-1 and macrophages polarization. Thus, we hypothesize that anesthetics may reduce M1 macrophage infiltration, potentially through the upregulation of these four genes, thereby providing cardioprotective effects. However, this hypothesis remains speculative and demands confirmation in future studies.

Nonetheless, this study also has several limitations that should be acknowledged. First, although the public database GEO has been utilized, the sample size is still restricted and requires expansion. Therefore, further validation with larger sample sizes is crucial to further validate the expression levels of MCL1, RGS2, PPP1R15A, and MAFF in atrial or blood samples taken from patients receiving sevoflurane or propofol during heart surgery. Second, in this study, we employed an *in vitro* H/R model to mimic ischemia-reperfusion injury occurring during cardiac surgery. Our findings demonstrate that both sevoflurane and propofol can upregulate the expression of Maff, Ppp1r15a, Rgs2, and Mcl1 in cardiomyocytes following H/R injury, implying that these anesthetics may confer cardioprotection by modulating these genes. However, this potential mechanism warrants further investigation through genetic manipulation approaches, such as gene overexpression or knockdown, to establish a causal relationship. Third, future research could develop personalized anesthetic protocols based on patient-specific gene expression profiles (e.g., avoiding anesthetic agents that suppress MCL1 in patients with low MCL1 expression). Furthermore, subsequent investigations should further elucidate the molecular regulatory mechanisms of these genes. By developing specific small-molecule modulators targeting the expression or protein function of MCL1, RGS2, and other related genes, it may establish a novel cardioprotective therapeutic strategy to improve perioperative myocardial protection in cardiac surgery patients.

## 5 Conclusion

In this study, we identified that MCL1, RGS2, PPP1R15A, and MAFF may serve as the hub Mito-RGs related to anesthetics. These four genes may serve as potential biomarkers related to the protective effects of anesthetics.

## Data Availability

The original contributions presented in the study are included in the article/Supplementary Material, further inquiries can be directed to the corresponding author.

## Supplementary material

Supplementary Figure S1 Immune cell infiltration analysis for sevoflurane and propofol groups respectively. **(A)** Box plots showing levels of 22 types of immune cells between Post_sevoflurane and Pre_sevoflurane groups. **(B)** Box plots showing levels of 22 types of immune cells between Post_propofol and Pre_propofol groups.

Supplementary Table S1 Mitochondrial-related genes.

Supplementary Table S2 Differentially expressed genes.

Supplementary Table S3 The results of GO and KEGG functional analysis.

Supplementary Table S4 Four hub genes and their upstream miRNAs.

Supplementary Table S5 Differentially expressed TFs.

Supplementary Table S6 The relationship between four hub genes and TFs.

## References

[1] Liu S Li Y Zeng X Wang H Yin P Wang L Burden of cardiovascular diseases in China, 1990–2016: findings from the 2016 global burden of disease study. JAMA Cardiol. (2019) 4(4):342–52. 10.1001/jamacardio.2019.0295 30865215 PMC6484795

[2] Xiang Y Zhao Q Wang N Yu Y Wang R Zhang Y Association of obesity with the risk of hyperhomocysteinemia among the Chinese community residents: a prospective cohort study in Shanghai, China. Nutrients. (2021) 13(10):3648. 10.3390/nu13103648 34684648 PMC8537264

[3] Thuan PQ Chuong PTV Nam NH Dinh NH. Coronary artery bypass surgery: evidence-based practice. Cardiol Rev. (2023) 33(4):344–51. 10.1097/CRD.0000000000000621 38112423

[4] Bana A Sangal A Mehta N Jaiswal S Tirkey S Yadav VK Off-pump CABG surgery in left main coronary artery disease: a single-center prospective registry. Indian J Thorac Cardiovasc Surg. (2023) 39(5):446–52. 10.1007/s12055-023-01526-3 37609610 PMC10441988

[5] Ishak NH Chong SE Zainal Abidin H Mamat AZ Mokthar AM Dimon MZ. Off-pump coronary artery bypass grafting surgery: a valuable 2-day experience. Malays J Med Sci. (2022) 29(6):158–63. 10.21315/mjms2022.29.6.15 36818905 PMC9910366

[6] Shaefi S Mittel A Loberman D Ramakrishna H. Off-pump versus on-pump coronary artery bypass grafting-a systematic review and analysis of clinical outcomes. J Cardiothorac Vasc Anesth. (2019) 33(1):232–44. 10.1053/j.jvca.2018.04.012 29753665

[7] Khalil MA Kaddoura R Omar AS Abohamar AD Izham M. Optimum heparin dose in off-pump coronary artery bypass grafting: a systematic review and meta-analysis. Perfusion. (2024) 39(4):675–83. 10.1177/02676591231159506 36858479

[8] Wang X Wang X Liu J Zuo YX Zhu QM Wei XC Effects of ciprofol for the induction of general anesthesia in patients scheduled for elective surgery compared to propofol: a phase 3, multicenter, randomized, double-blind, comparative study. Eur Rev Med Pharmacol Sci. (2022) 26(5):1607–17. 10.26355/eurrev_202203_28228 35302207

[9] Lotz C Stumpner J Smul TM. Sevoflurane as opposed to propofol anesthesia preserves mitochondrial function and alleviates myocardial ischemia/reperfusion injury. Biomed Pharmacother. (2020) 129:110417. 10.1016/j.biopha.2020.110417 32574972

[10] Heybati K Zhou F Baltazar M Poudel K Ochal D Ellythy L Appraisal of postoperative outcomes of volatile and intravenous anesthetics: a network meta-analysis of patients undergoing cardiac surgery. J Cardiothorac Vasc Anesth. (2023) 37(11):2215–22. 10.1053/j.jvca.2023.07.011 37573213

[11] Milne B John M Evans R Robertson S Scanaill PÓ Murphy GJ Comparison between propofol and total inhalational anaesthesia on cardiovascular outcomes following on-pump cardiac surgery in higher-risk patients: a randomised controlled pilot and feasibility study. Open Heart. (2024) 11(1):e002630. 10.1136/openhrt-2024-002630 38724266 PMC11086547

[12] Wu J Cai W Du R Li H Wang B Zhou Y Sevoflurane alleviates myocardial ischemia reperfusion injury by inhibiting P2X7-Nlrp3 mediated pyroptosis. Front Mol Biosci. (2021) 8:768594. 10.3389/fmolb.2021.768594 34765646 PMC8576530

[13] Elgebaly AS Fathy SM Sallam AA Elbarbary Y. Cardioprotective effects of propofol-dexmedetomidine in open-heart surgery: a prospective double-blind study. Ann Card Anaesth. (2020) 23(2):134–41. 10.4103/aca.ACA_168_18 32275025 PMC7336971

[14] Abraham AS Elliott CW Abraham MS Ahuja S. Intra-operative anesthetic induced myocardial protection during cardiothoracic surgery: a literature review. J Thorac Dis. (2023) 15(12):7042–9. 10.21037/jtd-23-1101 38249920 PMC10797362

[15] Yang XL Wang D Zhang GY Guo XL. Comparison of the myocardial protective effect of sevoflurane versus propofol in patients undergoing heart valve replacement surgery with cardiopulmonary bypass. BMC Anesthesiol. (2017) 17(1):37. 10.1186/s12871-017-0326-2 28259141 PMC5336653

[16] Li F Yuan Y. Meta-analysis of the cardioprotective effect of sevoflurane versus propofol during cardiac surgery. BMC Anesthesiol. (2015) 15:128. 10.1186/s12871-015-0107-8 26404434 PMC4583176

[17] Dost B Turunc E Sarikaya Ozel E Aydin ME Karapinar YE Beldagli M Myocardial protection in cardiac surgery: exploring the influence of anesthetic agents. Eurasian J Med. (2023) 55(1):138–41. 10.5152/eurasianjmed.2023.23376 38752865 PMC11075016

[18] Peoples JN Saraf A Ghazal N Pham TT Kwong JQ. Mitochondrial dysfunction and oxidative stress in heart disease. Exp Mol Med. (2019) 51(12):1–13. 10.1038/s12276-019-0355-7 31857574 PMC6923355

[19] Zhang XX Wu XS Mi SH Fang SJ Liu S Xin Y Neuregulin-1 promotes mitochondrial biogenesis, attenuates mitochondrial dysfunction, and prevents hypoxia/reoxygenation injury in neonatal cardiomyocytes. Cell Biochem Funct. (2020) 38(5):549–57. 10.1002/cbf.3503 32037595

[20] Ollitrault P Allouche S Chequel M Milliez P Alexandre J. Plasma aldosterone and atrial mitochondrial functions of patients undergoing cardiac surgery. Future Cardiol. (2020) 16(4):275–80. 10.2217/fca-2019-0028 32286862

[21] Tan Y Li T Hu M Wang B Zhou Q Jiang Y Phlpp1 deficiency ameliorates cardiomyocyte death and cardiac dysfunction through inhibiting mcl-1 degradation. Cell Signal. (2022) 92:110281. 10.1016/j.cellsig.2022.110281 35151832

[22] Raveh A Schultz PJ Aschermann L Carpenter C Tamayo-Castillo G Cao S Identification of protein kinase C activation as a novel mechanism for Rgs2 protein upregulation through phenotypic screening of natural product extracts. Mol Pharmacol. (2014) 86(4):406–16. 10.1124/mol.114.092403 25086086 PMC6067637

[23] Xie Z Lin H Huang Y Wang X Lin H Xu M Bap1-mediated maff deubiquitylation regulates tumor growth and is associated with adverse outcomes in colorectal cancer. Eur J Cancer. (2024) 210:114278. 10.1016/j.ejca.2024.114278 39151323

[24] Monkley S Overed-Sayer C Parfrey H Rassl D Crowther D Escudero-Ibarz L Sensitization of the upr by loss of Ppp1r15a promotes fibrosis and senescence in ipf. Sci Rep. (2021) 11(1):21584. 10.1038/s41598-021-00769-7 34732748 PMC8566588

[25] Zhang T Nie Y Gu J Cai K Chen X Li H Identification of mitochondrial-related prognostic biomarkers associated with primary bile acid biosynthesis and tumor microenvironment of hepatocellular carcinoma. Front Oncol. (2021) 11:587479. 10.3389/fonc.2021.587479 33868990 PMC8047479

[26] Ritchie ME Phipson B Wu D Hu Y Law CW Shi W Limma powers differential expression analyses for rna-sequencing and microarray studies. Nucleic Acids Res. (2015) 43(7):e47. 10.1093/nar/gkv007 25605792 PMC4402510

[27] Yu G Wang LG Han Y He QY. Clusterprofiler: an R package for comparing biological themes among gene clusters. OMICS. (2012) 16(5):284–7. 10.1089/omi.2011.0118 22455463 PMC3339379

[28] Newman AM Liu CL Green MR Gentles AJ Feng W Xu Y Robust enumeration of cell subsets from tissue expression profiles. Nat Methods. (2015) 12(5):453–7. 10.1038/nmeth.3337 25822800 PMC4739640

[29] Huang M Huiskes FG de Groot NMS Brundel B. The role of immune cells driving electropathology and atrial fibrillation. Cells. (2024) 13(4):311. 10.3390/cells13040311 38391924 PMC10886649

[30] Moscarelli M Punjabi PP Miroslav GI Del Sarto P Fiorentino F Angelini GD. Myocardial conditioning techniques in off-pump coronary artery bypass grafting. J Cardiothorac Surg. (2015) 10:7. 10.1186/s13019-014-0204-7 25599579 PMC4304196

[31] Xie D Deng H Feng H. Sevoflurane exerts improved protective effects than propofol on hypoxia-reoxygenation injury by regulating the microrna-221-5p/Adam8 axis in cardiomyocytes. Exp Ther Med. (2021) 22(2):893. 10.3892/etm.2021.10325 34257708 PMC8243314

[32] Zhang J Yu P Hua F Hu Y Xiao F Liu Q Sevoflurane postconditioning reduces myocardial ischemia reperfusion injury-induced necroptosis by up-regulation of ogt-mediated O-glcnacylated Ripk3. Aging (Albany NY). (2020) 12(24):25452–68. 10.18632/aging.104146 33231560 PMC7803485

[33] Zhang Q Cai S Guo L Zhao G. Propofol induces mitochondrial-associated protein lrpprc and protects mitochondria against hypoxia in cardiac cells. PLoS One. (2020) 15(9):e0238857. 10.1371/journal.pone.0238857 32898195 PMC7478836

[34] Xie H Zhang J Zhu J Liu LX Rebecchi M Hu SM Sevoflurane post-conditioning protects isolated rat hearts against ischemia-reperfusion injury via activation of the Erk1/2 pathway. Acta Pharmacol Sin. (2014) 35(12):1504–13. 10.1038/aps.2014.78 25345742 PMC4261124

[35] Sakuma Y Hirai S Sumi T Tada M Kojima T Niki T Mcl1 inhibition enhances the efficacy of docetaxel against airway-derived squamous cell carcinoma cells. Exp Cell Res. (2021) 406(2):112763. 10.1016/j.yexcr.2021.112763 34358524

[36] Pereira-Castro I Garcia BC Curinha A Neves-Costa A Conde-Sousa E Moita LF Mcl1 alternative polyadenylation is essential for cell survival and mitochondria morphology. Cell Mol Life Sci. (2022) 79(3):164. 10.1007/s00018-022-04172-x 35229202 PMC11072748

[37] Thomas RL Gustafsson AB. Mcl1 is critical for mitochondrial function and autophagy in the heart. Autophagy. (2013) 9(11):1902–3. 10.4161/auto.26168 24165322 PMC4028340

[38] Zheng Y Wei W Wang Y Li T Wei Y Gao S. Gypenosides exert cardioprotective effects by promoting mitophagy and activating Pi3k/akt/gsk-3beta/mcl-1 signaling. PeerJ. (2024) 12:e17538. 10.7717/peerj.17538 38912051 PMC11193969

[39] Zhang W Ren H Xu C Zhu C Wu H Liu D Hypoxic mitophagy regulates mitochondrial quality and platelet activation and determines severity of I/R heart injury. Elife. (2016) 5:e21407. 10.7554/eLife.21407 27995894 PMC5214169

[40] Wright T Turnis ME Grace CR Li X Brakefield LA Wang YD Anti-apoptotic mcl-1 promotes long-chain fatty acid oxidation through interaction with Acsl1. Mol Cell. (2024) 84(7):1338–53.e8. 10.1016/j.molcel.2024.02.035 38503284 PMC11017322

[41] Dong Z Zhao P Xu M Zhang C Guo W Chen H Astragaloside iv alleviates heart failure via activating pparalpha to switch glycolysis to fatty acid Beta-oxidation. Sci Rep. (2017) 7(1):2691. 10.1038/s41598-017-02360-5 28578382 PMC5457407

[42] Deng Y Dickey JE Saito K Deng G Singh U Jiang J Elucidating the role of Rgs2 expression in the pvn for metabolic homeostasis in mice. Mol Metab. (2022) 66:101622. 10.1016/j.molmet.2022.101622 36307046 PMC9638802

[43] Milanesi E Cucos CA Matias-Guiu JA Pinol-Ripoll G Manda G Dobre M Reduced blood Rgs2 expression in mild cognitive impairment patients. Front Aging Neurosci. (2021) 13:738244. 10.3389/fnagi.2021.738244 34658840 PMC8513788

[44] Hadar A Milanesi E Squassina A Niola P Chillotti C Pasmanik-Chor M Rgs2 expression predicts amyloid-Beta sensitivity, mci and alzheimer’s disease: genome-wide transcriptomic profiling and bioinformatics data mining. Transl Psychiatry. (2017) 7(2):e1035. 10.1038/tp.2016.228 28195566 PMC5438017

[45] Nunn C Zou MX Sobiesiak AJ Roy AA Kirshenbaum LA Chidiac P. Rgs2 inhibits Beta-adrenergic receptor-induced cardiomyocyte hypertrophy. Cell Signal. (2010) 22(8):1231–9. 10.1016/j.cellsig.2010.03.015 20362664

[46] Jiang L Xu L Zheng L Wang Y Zhuang M Yang D. Salidroside attenuates sepsis-associated acute lung injury through Ppp1r15a mediated endoplasmic Reticulum stress inhibition. Bioorg Med Chem. (2022) 71:116865. 10.1016/j.bmc.2022.116865 35985062

[47] Ito S Tanaka Y Oshino R Okado S Hori M Isobe KI. Gadd34 suppresses lipopolysaccharide-induced sepsis and tissue injury through the regulation of macrophage activation. Cell Death Dis. (2016) 7(5):e2219. 10.1038/cddis.2016.116 27171261 PMC4917654

[48] Hayakawa-Ogura M Tana Nakagawa T Itoh M. Gadd34 suppresses Eif2alpha phosphorylation and improves cognitive function in Alzheimer’s disease-model mice. Biochem Biophys Res Commun. (2023) 654:112–9. 10.1016/j.bbrc.2023.02.077 36907138

[49] Zhou G Peng Y Guo M Qu C Luo S Jiang Y Esomeprazole inhibits endoplasmic Reticulum stress and ameliorates myocardial ischemia-reperfusion injury. Biochem Biophys Res Commun. (2022) 627:84–90. 10.1016/j.bbrc.2022.08.013 36030656

[50] Toro R Perez-Serra A Mangas A Campuzano O Sarquella-Brugada G Quezada-Feijoo M Mir-16-5p suppression protects human cardiomyocytes against endoplasmic reticulum and oxidative stress-induced injury. Int J Mol Sci. (2022) 23(3):1036. 10.3390/ijms23031036 35162959 PMC8834785

[51] von Scheidt M Zhao Y de Aguiar Vallim TQ Che N Wierer M Seldin MM Transcription factor maff (maf basic leucine zipper transcription factor F) regulates an atherosclerosis relevant network connecting inflammation and cholesterol metabolism. Circulation. (2021) 143(18):1809–23. 10.1161/CIRCULATIONAHA.120.050186 33626882 PMC8124091

[52] Wang M Liu F Fang B Huo Q Yang Y. Proteome-scale profiling reveals maff and mafg as two novel key transcription factors involved in palmitic acid-induced umbilical vein endothelial cell apoptosis. BMC Cardiovasc Disord. (2021) 21(1):448. 10.1186/s12872-021-02246-5 34535081 PMC8447594

[53] Ibrahim MK Abdelhafez TH Takeuchi JS Wakae K Sugiyama M Tsuge M Maff is an antiviral host factor that suppresses transcription from hepatitis B virus core promoter. J Virol. (2021) 95(15):e0076721. 10.1128/JVI.00767-21 33980595 PMC8274605

[54] Lei D Wang Y Li S Xiang S Luo Y Yan P Maff alleviates hepatic ischemia-reperfusion injury by regulating the Clcf1/Stat3 signaling pathway. Cell Mol Biol Lett. (2025) 30(1):39. 10.1186/s11658-025-00721-x 40169936 PMC11963299

[55] Li B Yu J Liu P Zeng T Zeng X. Astragaloside iv protects cardiomyocytes against hypoxia injury via hif-1alpha and the Jak2/Stat3 pathway. Ann Transl Med. (2021) 9(18):1435. 10.21037/atm-21-4080 34733987 PMC8506767

[56] Nakao S Tsukamoto T Ueyama T Kawamura T. Stat3 for cardiac regenerative medicine: involvement in stem cell biology, pathophysiology, and bioengineering. Int J Mol Sci. (2020) 21(6):1937. 10.3390/ijms21061937 32178385 PMC7139789

[57] Cen M Ouyang W Zhang W Yang L Lin X Dai M Mitoq protects against hyperpermeability of endothelium barrier in acute lung injury via a Nrf2-dependent mechanism. Redox Biol. (2021) 41:101936. 10.1016/j.redox.2021.101936 33752110 PMC8005834

[58] Tung B Xia S. Kruppel-like factor 4 (Klf4) and its regulation on mitochondrial homeostasis. J Stem Cell Res Ther. (2018) 8(9):436. 10.4172/2157-7633.1000436 31245170 PMC6594694

[59] Li Y Xiong Z Jiang Y Zhou H Yi L Hu Y Klf4 deficiency exacerbates myocardial ischemia/reperfusion injury in mice via enhancing Rock1/Drp1 pathway-dependent mitochondrial fission. J Mol Cell Cardiol. (2023) 174:115–32. 10.1016/j.yjmcc.2022.11.009 36509022

[60] Wang J Zhao M Zhang H Yang F Luo L Shen K Klf4 alleviates hypertrophic scar fibrosis by directly activating Bmp4 transcription. Int J Biol Sci. (2022) 18(8):3324–36. 10.7150/ijbs.71167 35637963 PMC9134901

[61] Topal D Korkmaz UTK Velioglu Y Yuksel A Donmez I Ucaroglu ER Systemic immune-inflammation index as a novel predictor of atrial fibrillation after off-pump coronary artery bypass grafting. Rev Assoc Med Bras (1992). (2022) 68(9):1240–6. 10.1590/1806-9282.20220295 36228255 PMC9575030

[62] Urbanowicz T Michalak M Al-Imam A Olasinska-Wisniewska A Rodzki M Witkowska A The significance of systemic immune-inflammatory Index for mortality prediction in diabetic patients treated with off-pump coronary artery bypass surgery. Diagnostics (Basel). (2022) 12(3):634. 10.3390/diagnostics12030634 35328187 PMC8947274

[63] Huang L Duan F Dong X Zhang Z. The N6-methyladenosine pattern of Map3k7 mediates the effects of sevoflurane on macrophage M2 polarization and cervical cancer migration and invasion. Cent Eur J Immunol. (2024) 49(4):393–403. 10.5114/ceji.2024.145307 39944262 PMC11811724

[64] Yu F Bai T. Sevoflurane activates the il-6/ho-1 pathway to promote macrophage M2 polarization and prostate cancer lung metastasis. Int Immunopharmacol. (2022) 113(Pt A):109380. 10.1016/j.intimp.2022.109380 36330914

[65] Liu Z Meng Y Miao Y Yu L Wei Q Li Y Propofol ameliorates renal ischemia/reperfusion injury by enhancing macrophage M2 polarization through ppargamma/Stat3 signaling. Aging (Albany NY). (2021) 13(11):15511–22. 10.18632/aging.203107 34111028 PMC8221315

[66] Yang H Chen Y Gao C. Interleukin-13 reduces cardiac injury and prevents heart dysfunction in viral myocarditis via enhanced M2 macrophage polarization. Oncotarget. (2017) 8(59):99495–503. 10.18632/oncotarget.20111 29245918 PMC5725109

[67] Fan Q Tao R Zhang H Xie H Lu L Wang T Dectin-1 contributes to myocardial ischemia/reperfusion injury by regulating macrophage polarization and neutrophil infiltration. Circulation. (2019) 139(5):663–78. 10.1161/CIRCULATIONAHA.118.036044 30586706

[68] Mouton AJ Li X Hall ME Hall JE. Obesity, hypertension, and cardiac dysfunction: novel roles of immunometabolism in macrophage activation and inflammation. Circ Res. (2020) 126(6):789–806. 10.1161/CIRCRESAHA.119.312321 32163341 PMC7255054

[69] Tan C Li C Ge R Zhang W Wu Z Wang S Mcl-1 downregulation enhances bcg treatment efficacy in bladder cancer by promoting macrophage polarization. Cancer Cell Int. (2025) 25(1):48. 10.1186/s12935-025-03676-3 39955585 PMC11830210

