# Supplementary figures and images for: Identification of mitochondria-related genes associated with anesthetics in patients undergoing off-pump coronary artery bypass grafting surgery
Source: Front Surg. 2025 Jul 9;12:1515732. doi: 10.3389/fsurg.2025.1515732 (PMC12283725; doi:10.3389/fsurg.2025.1515732)

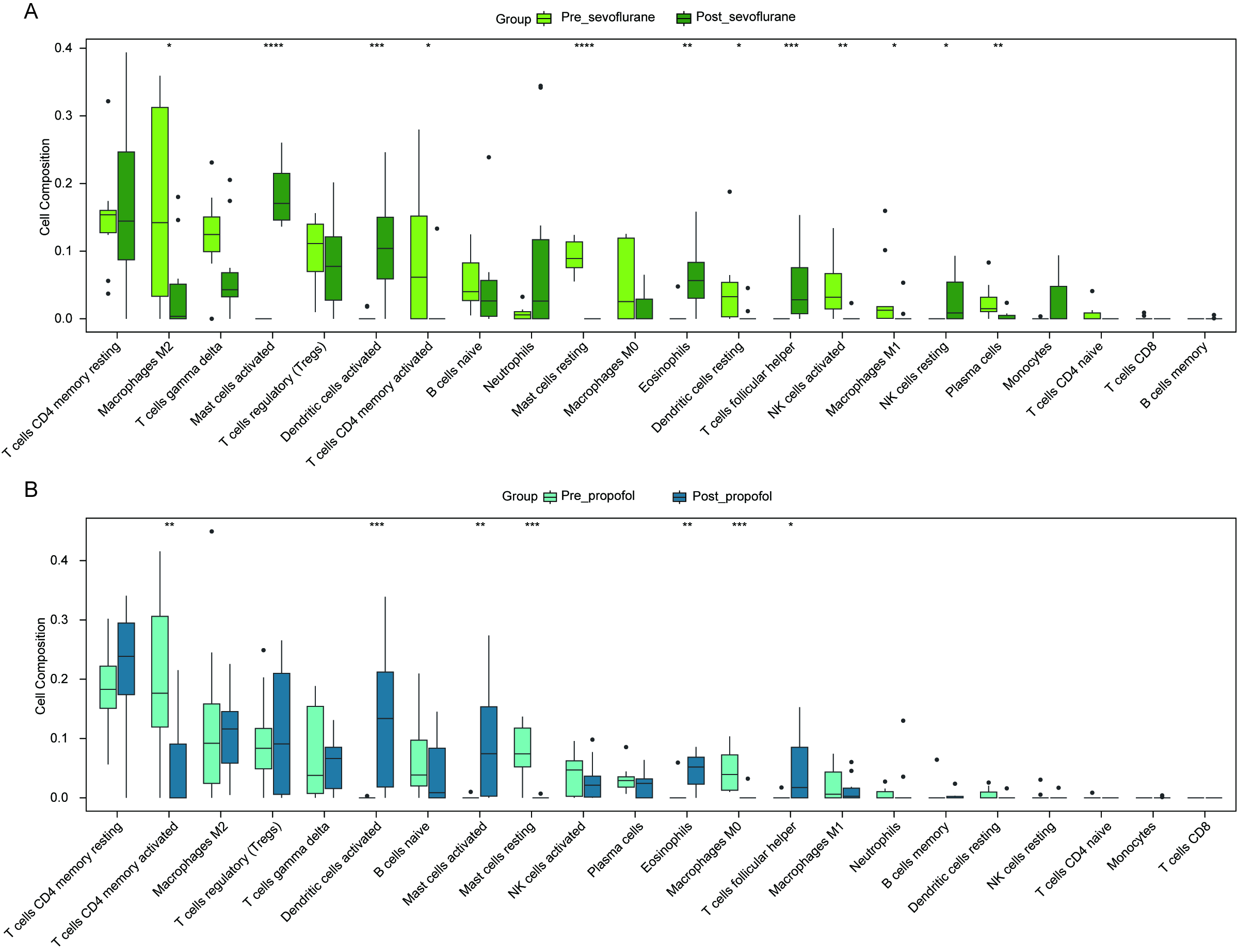

Supplement: Supplementary Figure S1 — Immune cell infiltration analysis for sevoflurane and propofol groups respectively. (A) Box plots showing levels of 22 types of immune cells between Post_sevoflurane and Pre_sevoflurane groups. (B) Box plots showing levels of 22 types of immune cells between Post_propofol and Pre_propofol groups. [file Image1.tif]
